# Social behaviors as welfare indicators in teleost fish

**DOI:** 10.3389/fvets.2023.1050510

**Published:** 2023-04-24

**Authors:** Luciano Cavallino, Laura Rincón, María Florencia Scaia

**Affiliations:** ^1^Instituto de Biodiversidad y Biología Experimental y Aplicada – CONICET, Ciudad Autónoma de Buenos Aires, Argentina; ^2^Laboratorio de Neuroendocrinología y Comportamiento, Departamento de Biodiversidad y Biología Experimental, Facultad de Ciencias Exactas y Naturales, Universidad de Buenos Aires, Ciudad Autónoma de Buenos Aires, Argentina

**Keywords:** social behavior, welfare indicators, cichlid fish, Cypriniformes, Characiformes, cortisol, sexual steroids

## Abstract

Animal welfare is a key issue not only for aquaculture industry and food production, but also for daily husbandry practices in research topics related to physiology in wild and farmed animals. In this context, teleost fish constitute interesting models to assess alternative welfare indicators because of their wide diversity in reproductive and social structures. Any framework for assessing teleost fish welfare needs to account for the physiological mechanisms involved in each species as a first step. A comprehensive approach should also take into account how these physiological and behavioral parameters can be altered by environmental enrichment considering the specific requirements in each case and identifying intrinsic biological characteristics of individual species. This review will show how cortisol and sex steroids regulate social behavior in teleost fish, and how different aspects of social behavior can be employed as welfare indicators according to specific characteristics in each case. This article will consider evidence in teleost fish, including cichlids, characids and cyprinids with different reproductive strategies and social structures (e.g., territorial social hierarchies or shoaling behavior). Neotropical species will be particularly emphasized. The main laboratory-based animal welfare indicators are cortisol, a classical stress hormone, together with sex steroids. Considering that the endocrine landscape is intrinsically related to social behavior, reproductive and agonistic behavioral traits such as aggression, anxiety and courtship are key elements to assess welfare under housing and culture conditions. This review highlights the importance of assessing physiological mechanisms and identifying behavioral characteristics in teleost fish, especially in Neotropical species, as a baseline to understand which environmental enrichment can improve animal welfare in each individual species.

## 1. Introduction

Human activities involving live animals may affect welfare in different ways and, even if husbandry standards and housing requirements have been established for different groups, a better empirical understanding of animal welfare is continuously required. Many domestic animals or those used for food production are frequently kept under confined conditions in which basic behavioral and physiological needs are drastically affected. Taking into account the continually growing anthropocentric activities involving the use of animals for research, medicinal use and food production, animal welfare represents a central area of growing interest. An increasing number of human activities can potentially affect fish welfare, including environmental degradation, farming, commercial fisheries, aquaculture, ornamental fish, sports fishing, and scientific research (1). A better understanding of fish welfare will increase awareness in groups leading these activities and to this end a thorough revision of welfare indicators will help to understand the biological requirements for particular fish species.

Specific indicators need to assess potential welfare problems and give information on the internal state of animals exposed to human activities (2). While some welfare indicators can be easily sampled in natural environment and in farms, others can be more difficult to apply on site since they can involve laboratory analysis or a specific analytic facility (3). This way, behavioral welfare indicators are very good candidates to use “on-farm,” since they are easy to observe and can be used as a non-invasive approach to assess any potential welfare problem (4). Behavior involves fish reaction to their perception of the environment and behavioral variables are key aspects to take into account when assessing their welfare and global internal state (5). Some specific behavioral traits are widely used as welfare indicators, such as food intake, changes in food anticipatory behavior, swimming activity, and ventilation rate (1). However, other behaviors are less used as welfare indicators, such as social, reproductive, and parental behaviors. Considering that behavior is modulated by physiology and motivational states of each individual fish, hormonal and neuroendocrine regulation play central roles in social and reproductive behaviors. Even if behavioral responses are very useful indicators, a robust and integrated welfare assessment also requires other factors such as endocrine, neurophysiology, pathological, and molecular tools (1). In this intertwined relationship between behavior, hormones and animal welfare, it is important to distinguish the endocrine mechanisms regulating reproductive and social behavior, in order to identify specific behavioral displays that can be used as welfare indicators. In this sense, sex steroids and cortisol play central roles modulating agonistic behaviors such as aggressive and submissive displays, courtship, mating, and parental behaviors (6, 7).

Taking into account that teleosts are the largest and most diverse group of vertebrates, with more than 30,000 species described (8), they represent very interesting biological models to assess welfare indicators. Teleost fish constitute the dominant evolutionary radiation of vertebrates in our planet, which is reflected not only in the vast array of colonized habitats, but also in a remarkable variety of ecological specializations, physiological mechanisms and behavioral characteristics. Considering the popular use of some specific teleost species in human activities, these examples illustrate the diversity in physiological mechanisms underlying behavior and the need to use this species-specific information as welfare indicators. It is estimated that 90% of the total volume of trade in ornamental fish corresponds to tropical freshwater fish (9). Considering this, this review will focus on specific teleost groups based not only on their relevance in human economic activities and the potential impact on their animal welfare, but also on the current knowledge on their behavioral strategies and biology.

Cichlid fish are a very appealing family to discuss the importance of behavioral traits as welfare indicators, since they show diverse social networks with hierarchical structures and different parental care or reproductive strategies. In most species with social hierarchies, dominants aggressively defend a spawning territory from subordinate, nonterritorial fish (10). While some species are important for food production and aquaculture activities, others are considered game fish and have been introduced to regions beyond their natural habitat, usually linked to escapes or releases from fish farms. Based on their native range, cichlids can be classified as African and Neotropical cichlids. Neotropical region is comprised by North, Central and South America and includes 67 described genus (11). While African cichlids usually inhabit lakes, Neotropical cichlids can be mainly found in lentic habitats such as rivers (12). Neotropical species have a central role in aquaculture and, as a consequence, their welfare can be deeply affected. Interestingly, the most studied species to date are the African cichlids, while Neotropical species are understudied despite of their economic relevance. Characiforms also include African and Neotropical species living in rivers and lakes. They also present different reproductive strategies and social behavior ranging from aggressive species such as piranhas to shoaling and/or schooling species such as tetras (13). Some characiforms are very popular in the aquarium trade (tetras, piranhas, and pencilfishes), while others have a commercial importance as sport-fishing species (the African tiger fish and the Neotropical dorados) or as food resource for human communities living near tropical rivers (the African citharinids and the Neotropical prochilodontids). Cypriniformes also includes species with very important scientific and commercial value in aquaculture, breeding, food industry and ornamental fisheries, such as carps, minnows, goldfish and zebrafish. Even if wild stocks can be found in different continents depending on each case, these species have been deeply introduced in diverse environments (14).

The aim of this review is to highlight the importance of studying behavior to assess the welfare of different teleost fish. With this main focus, we review physiological mechanisms underlying complex social behaviors, emphasizing the central role of cortisol as the main stress parameter affecting fish social structure and sex steroids regulating reproductive and parental behavioral strategies. Throughout this review we intend to highlight the need to understand the physiological mechanisms and the behavioral characteristics of different teleost fish according to their specific biological requirements, with the perspective of using behavioral traits as welfare indicators. Because of their behavioral curiosities and their intense use by human activities, we will focus on different examples of cichlid fish, characiforms, and cypriniforms.

## 2. Teleost fish as biological models for welfare indicators

Considering that multiple human activities involve teleost fish, there is a growing body of evidence suggesting that human activities can harm their welfare (1). However, the magnitude of these effects depends on the general characteristics or life-history of each group and with this in mind, it is important to revise behavioral and physiological mechanisms explaining how to analyze welfare indicators in each species.

Cichlid fish are very popular in aquaculture industry, and they constitute ideal models to understand reproductive physiology and neuroendocrine basis of social behavior. Considering their complex social structure, cichlids show different reproductive strategies and diverse forms of parental care, such as substrate guarding and mouthbrooding, and also an impressive variation in which sex provides parental care (e.g., biparental, female-only, or male-only care). The African cichlid fish radiations are the most diverse animal radiations, with most species endemic to specific lakes such as the Lake Malawi, Lake Victoria, and Lake Tanganyika (15). The most popular cichlid models to study the neuroendocrine mechanisms regulating social behavior and reproduction are the African species *Astatotilapia burtoni*, *Neolamprologus pulcher*, and *Oreochromis mossambicus*. Considering that freshwater fish species dominate aquaculture fish production, with carps and cyprinids representing over 53% of fish production, it is important to emphasize that cichlids constitute 11% of this production (16). The most popular cichlids are tilapias, in particular Nile tilapia (*Oreochromis niloticus*) and its hybrids accounting for over 90% of production of tilapias (17). This species is not only widely used for neurobiological and endocrine studies, but it is also globally important as a cultured food fish since it is one of the most important fish species in worldwide aquaculture. Uncontrolled breeding of tilapias led to excessive recruitment, high densities, stunting and low percentage of marketable-size fish, until research on nutrition, culture systems and the development of hormonal sex-reversal techniques in 1970 allowed for monosex populations to be raised in uniform sizes (18). Consistently, most of the evidence assessing animal welfare in cichlids refers to *O. niloticus,* usually regarding welfare indicators of stress (19) or dietary impacts on improving the antioxidant status and general health in this species (20). However, evidence on welfare indicators in other cichlids is scarce. Therefore, it is very important to revise physiological mechanisms underlying the behavioral strategies of different species, in order to assess the importance of different behavioral traits as welfare indicators.

Cypriniformes includes more than 3,000 species and almost all of them are strictly freshwater and distributed across Eurasia, Africa, and North America (14). Besides carps and cyprinids, representing over 53% of fish production, the most important ornamental fishes are goldfish (*Carassius auratus*). Originally from southern Asia, they were later introduced in a broad range of freshwater habitats worldwide (21). As one of the most important cases in aquaculture, this species has been one of the first animals bred with an ornamental purpose (22) and it is widely used as a biological model in research on different fields such as endocrinology, developmental biology, fish pathology among others. Another species with economic importance is the common carp (*Cyprinus carpio*) native to Europe and Asia, but due to its invasive capacity it is found in many countries and freshwater habitats around the world, mainly ponds, lakes, and rivers (23). Because of its popularity as an ornamental fish, the commercial production of carp increased considerably (24), while it has also been used in scientific research in different fields such as genetic research, developmental biology, immunological systems, studies of tissue damage and the effect of different diets on growth rates and health. Zebrafish (*Danio rerio*) constitutes a particular case among Cypriniformes. Even if it is also traded as an ornamental fish, its main popularity worldwide is due to the fact that it is one of the most important non-mammalian biological models for scientific research in different fields such as genetics, behavioral genetics, endocrinology, memory and behavior, development, pathologies and as a model for psychiatric disorders and drug screening (25). Originally endemic to rivers and streams in India, Nepal, and Bangladesh (26), it has been easily introduced worldwide to breeding systems in laboratory conditions. Due to its importance as a model in experimental research, welfare indicators in this species were broadly studied. In particular, the main focus has been placed on behaviors related to stress and anxiety (27), poor living conditions and behavioral displays (28).

Characiformes is a group of freshwater fish with about 18 families and almost 2,000 species, very popular in the ornamental fish trade and commonly farmed in different countries (29). They live in tropical waters from the north of Patagonia in Argentina to the south of the United States, and this wide variety of environments is reflected in their great ecological and morphological diversity (30). Characidae is the most diverse family among Neotropical fishes and the fourth most diverse worldwide (31). The best-known representatives of Characiformes are the tetras, whose cultured stocks are usually domesticated (32) and constitute one of the most popular fishes kept in modern aquariums. Tetras are sociable, non-aggressive species and represent no threat to other fish, making them ideal for a community aquarium (33).

Popularity of ornamental fish has grown by 14% annually since the 1970s, and nowadays more than one billion individual fish are internationally traded each year (9). For example, neon tetra (*Paracheirodon innesi*) and cardinal tetra (*Paracheirodon axelrodi*) are two species of great interest in the ornamental trade (34). *P. innesi* is more commercialized than *P. axelrodi,* since it is much less sensitive in terms of management and reproduction in captivity. These species are collected from the wild in South America (35) and between 12 and 15 million neon tetras are annually exported, which represents about 80% of the total ornamental fish market in the state of Amazonas (9). Considering that intensive fishing can unbalance the populations in the natural environment, this situation has raised deep concern. However, despite their economic relevance, ornamental fish still face critical welfare challenges, including inadequate maintenance conditions, poor water quality, handling, transportation, confinement, overcrowding, poor diet, or feeding methods and disease (36). Even though research on characiforms welfare is scarce, exploring different indicators in this order is crucial to improve the quality of life and health of fish, and to expand successful production in the ornamental trade.

## 3. Social behavior

Social behavior, such as courtship, mating and aggression, involves individuals interacting with others and adjusting their behavior based upon previous experience in a constantly changing environment. Fish can present different social strategies, from shoaling and schooling to social hierarchies with dominant and subordinate individuals. Establishing dominance implies high levels of aggression and agonistic interactions before and during the development of social hierarchies. As a consequence, social stress during this process is one of the major welfare issues when rearing and keeping fish in captivity. Also reproductive behavior in fish includes very variable strategies such as monogamy (permanent or seasonal), polygamy (polygenic or polyandric) and specific systems like lek-breeding, harems and sneaking behaviors. This great variety of social behavioral strategies are represented in different examples of cichlid fish, Characiformes, and Cypriniformes.

These social displays and species-specific social behavioral strategies need to be taken into consideration when establishing requirements and housing conditions such as tank size, fish density, environmental enrichment, and diet. Many of these social behavioral traits are drastically affected when stocking densities are too low or too high, impacting directly on fish welfare. Since stocking density is a very important topic in aquaculture, an integrative approach should consider fish welfare and farmer’s perspectives by integrating solid welfare assessment (operational welfare indicators) with good management practices (37). Moreover, considering that social behavior can be affected by the environmental enrichment and that in most cases this focuses on structural enrichment, it is important to emphasize that many other strategies are essential to improve farmed fish welfare, such as sensorial (visual, auditory, and chemical stimuli), occupational enrichment (aiming to introduce diverse challenges to the rearing environment to prevent monotony), social and dietary enrichment [reviewed in (38)].

Cichlids typically form hierarchical social systems with one or two dominant animals defending their status from other submissive and lower-ranked animals (39). African species have been widely studied as biological models to assess the neuroendocrine mechanisms regulating social behavior and reproductive strategies. In *A. burtoni* one dominant male mates with several females and defends the territory by aggressive displays (40). Parental care in this species is typically female mouthbrooding and even if females have been traditionally described as non-aggressive, female aggression has been observed in all-female communities (41) and also in recently collected stock of fish females taking care of their brood (42). Behavioral differences between *A. burtoni* stocks have been attributed to the fact that the majority of traditional research in this species has been focused on a single fish stock collected from Lake Tanganyika in 1977, which has been maintained in captivity under laboratory conditions for more than 30 years. Artificial selection probably occurred in laboratory stocks, and this can explain differences in aggression observed in female wild stocks (42). This constitutes a clear example of the importance of using social behavior as welfare indicators, and it emphasizes the relevance of assessing social behavior in freshly collected wild stocks.

Another widely studied African cichlid species is *O. mossambicus* (Mozambique tilapia), a maternal mouthbrooding cichlid in which dominant males dig nests and court females, while subordinates wait for opportunities for social ascension or sneaking fertilization (43). Interestingly, dominant males can also display courtship behavior toward subordinates, who performed typical female sexual behavior (43). This male–male courtship constitutes another clear example of the importance of studying social behavior as welfare indicator in fish, since authors discuss that this behavior can be argued to be an effect of crowding under captive conditions. In *O. niloticus* high levels of aggression have been described during hierarchy formation (44) and also in specific aquaculture and laboratory conditions, even leading to high mortalities (45). Interestingly, cold water reduces aggression and it can be applied to mitigate adverse social effects arising from hierarchy formation in captive conditions (46).

On the contrary, *N. pulcher* is a cooperative breeding species with a dominant breeding pair showing aggressive behaviors and subordinate helpers that assist in taking care of the offspring (47, 48). Even if this species does not constitute a relevant example in worldwide aquaculture, aggression has been assessed as a welfare indicator in terms of other anthropogenic activities and stressors in aquatic environments, suggesting that underwater noise produced by commercial shipping and recreational boating reduces digging behavior and defense against predators, while it increases the amount of aggressive behavior (49).

Neotropical cichlid species are also characterized by complex social behaviors. Interestingly, while most African species present uniparental care of eggs and larvae usually associated to mouthbrooding behavior, Neotropical cichlids usually present biparental behavior (50). This is the case of *Cichlasoma dimerus*, a South American species inhabitant of the Parana and Paraguay Rivers’ basins that has been used as laboratory model to study the neuroendocrine basis of social behavior and reproduction (51). This is a serially monogamous species in which individuals display aggressive and submissive behaviors during the establishment and posterior maintenance of a very clear social hierarchy and, like most American cichlids, it is a substrate brooder with biparental behavior (6, 10). Aggressive behavior in this species has been characterized and quantified in different ethological contexts and, in all cases, males and females of this species show very high levels of aggressive and submissive displays, emphasizing the importance of this species as a biological model to study neuroendocrine basis of reproductive and social behavior (39).

Similar to *C. dimerus*, in Neotropical cichlids monogamous behavior and biparental care of the offspring are very common, so both males and females usually display aggressive behavior to defend their territory (52, 53). Moreover, the typical fighting behavior found in *C. dimerus* (54) is also observed in different Neotropical cichlid species, such as *Cichlasoma paranaense* (55) and *Astronotus ocellatus* (56). Even if these cases do not represent relevant commercial species, the study of social behavior results in a deeper knowledge on aggressive displays. The capacity of using this behavior as a welfare indicator is further discussed in the following section.

When assessing fish welfare, water renewal and temperature are key factors to take into account. Since chemical cues play a central role in communicating social status and regulating behavioral interactions among fish, water removal can wash out chemical substances and interfere in social rank signaling in highly social cichlids (57). In *C. paranaense*, water removal does not increase aggressive interactions, in contrast with evidence in *P. scalare*, suggesting interspecific differences on the aggressive response to chemical signaling in the social hierarchy (58, 59). Changes in water temperature can also alter aggressive behavior in a species-specific manner, since a raise in temperature increases aggressive displays in *Apistogramma agassizii* and *Amatitlania nigrofasciata* (60, 61), but *C. paranaense* reduces aggression in lower temperatures (62). These evidences show differences in how aggression can be interpreted as a welfare indicator and highlights species-specific requirements to ensure optimal social conditions.

Another relevant issue involving fish welfare is social isolation, which can be assessed in different cichlid species by studying aggression as a behavioral welfare indicator. Since cichlid fish are considered as very aggressive, they are usually kept in isolation in the aquaculture industry, such as *A. nigrofasciata* and the oscar *Astronotus ocellatus*. In these species, isolation increases the frequency of mouth fighting and the motivation for aggressive behavior (56, 63). However, isolation in cichlids impairs welfare not only in reference to aggression but in multiple ways, such as locomotor and feeding activity in *P. scalare* (64), the neuroendocrine system in *A. burtoni* (65) and also the cognitive performance in *C. paranaense* (55). This way, even if the consequences of isolation on welfare have been widely studied and have been shown to affect aggression in these specific cases, this may be also important in other cichlids and further research needs to assess the effect of social isolation using aggression as a behavioral welfare indicator.

Cypriniformes also have complex social structures and different behavioral traits can be very useful to assess fish welfare. While common carps are highly social and show aggressive behaviors like bites or chases, evidence suggests that high density induces higher aggression, swimming and feeding activity, while it reduces resting time (66). Aggression can be also used as an indicator in reference to fish exposure to heavy metal and other toxic compounds such as polyethylene microplastics, glyphosate (67) and cadmium (68). Zebrafish is a highly social species with shoaling behavior, in which both sexes establish social status by aggressive and submissive displays (69). Social status not only regulates access to reproduction, but also affects physiology and health of individuals in different ways, since subordinate fish show signals of immunosuppression and altered plasma levels of sex-steroid hormones (70). Social behavior can be a useful welfare indicator to assess the effect of overcrowding, inflammatory response and the exposure to specific substances, since ethanol and certain metals trigger antisocial behaviors in zebrafish (71, 72). Furthermore, exposure to conspecific alarm substances or caffeine alter the normal shoaling behavior in this species (73).

Regarding the reproductive behavior of cypriniform species mentioned here, males usually compete with each other to fertilize eggs and females can choose their mates, such as female zebrafish preferring larger males (74). In common carp, zebrafish and goldfish courtship is similar, with males chasing and encircling the female until eggs are released and they can be fertilized by, in some cases, several males simultaneously (75–77). Most of these species do not present parental care and, in fact, filial cannibalism is observed in some species (78). In zebrafish, egg cannibalism has been reported in field-based semi-natural or in laboratory conditions, and it has been suggested that females preferring gravel as a spawning substrate is related to the fact that eggs falling between gravel can be protected from disturbance or cannibalism (79). In this context, the question on whether filial cannibalism can be indicative of fish welfare can be raised. Evidence suggests that even if in some teleost species there is a decrease in cannibalism in males with supplementary feeding (80), manipulative studies in other species fail to support the prediction that well-fed parents would show decreased filial cannibalism than starved ones [discussed in (81)]. This way, even if egg cannibalism may be a welfare indicator in some species, in particular referring to the nutritional state, in other cases this behavior can be related to natural or seminatural conditions and to other factors such as parental behavior.

Despite the extraordinary diversity on breeding systems of teleost fish, social and reproductive behavior in Characiformes remain poorly studied in contrast to the closely related Cypriniformes and Siluriformes. One of the few characiform species in which the reproductive behavior has been formally studied is the sabalo *Prochilodus lineatus*, a Neotropical migratory fish with shoaling behavior during reproduction. DNA and multilocus genotyping methods suggest that this species shows a monogamous genetic mating system in combination with polygamy (82, 83). This knowledge on mating strategies and reproductive behavior of Neotropical migratory fishes in nature constitutes relevant evidence for the management and conservation of such an important fishery resource.

Within Characiformes, tetras are highly social species with schooling behavior. Fish aggregations can be classified as shoals, when there is a significant degree of cohesion among the individuals (e.g., social attraction), or as schools, when individuals perform synchronized behaviors in terms of speed and body orientation (84). In *P. innesi* robust schooling behavior is well reported (85), and evidence suggests that the individual optimal position in the group is context dependent since individual fish continuously modify their location in the school according to different external scenarios (13). In contrast to *P. innesi*, in *P. axelrodi* and the red-tailed tetra *Aphyocharax anisitsi* available information corresponds to collective knowledge from aquarists.

Considering that pheromones play a fundamental role in social communication, their importance in Characiformes has been assessed in two species. In swordtail characin *Corynopoma riisei,* evidence suggests that male sex pheromones from gill and anal fin glands induce behavioral changes in females, reducing stress and increasing female courtship behavior, thus facilitating reproduction (86, 87). In *A. anisitsi*, since gill glands are present in males with higher gonadosomatic index, they have been suggested as indicative of sex maturity (88). In this species evidence suggests that males respond to male and female chemical cues even in the absence of other sensory inputs (89). This evidence supports the possibility that pheromones participate in the chemical communication of *A. anisitsi* and is a clear example of how behavioral indicators can be used to assess not only social chemical cues in characids, but also exposure to aquatic environmental substances in characids (90).

## 4. Cortisol as classical stress parameter

Fish welfare can be affected by different common practices in aquaculture, such as overcrowding, handling, social isolation and transportation, all possible husbandry stressors affecting their quality of life (36, 91). Glucocorticoids are steroid hormones synthesized by the adrenal/interregnal gland, and cortisol is the main glucocorticoid in fish (92). Considering the stress-mediated increase in plasma glucocorticoids in all vertebrates, cortisol is the main component of the primary neuroendocrine stress response in fish. Stress can stimulate the hypothalamic–pituitary-interrenal (HPI) axis, inducing an increase in cortisol that affects brain functions, animals’ growth, reproduction, immune system and behavior in fish (93, 94). Unfortunately, different phases of the distribution market may result stressful for fish, and it has been reported that handling fish for even very short period of time (30s) is enough to increase cortisol in several species (95). On the other hand, different physiological markers of stress have been studied to assess the effect of short and long transport, including blood glucose, lactate and cortisol (96). Even if cortisol can be used as an indicator of how fish perceive their environment, social structure and aggressive interactions trigger high social stress in fish (93, 97). As a consequence, the use of cortisol as a welfare indicator in husbandry practices and culture environment needs to be assessed with caution considering physiological levels and social behavior characteristics of each species.

In highly social fish, hierarchies are a source of physiological and physical stress, since lower-ranked individuals are chronically stressed (98). This is observed in different cichlids, with species-specific peculiarities. In *A. burtoni*, non-territorial males show higher cortisol plasma levels than territorial males (99) and the same tendency is observed in *C. dimerus* (6, 100). However, in *N. pulcher* dominant males show higher cortisol than subordinates (101), while in *O. niloticus* territorial and non-territorial males present similar cortisol levels (102). This evidence illustrate the complex relationship between dominance and glucocorticoid levels in vertebrates, which varies among species but also depends on different characteristics of each social hierarchy, such as stability, environmental conditions, the type of breeding system and how the social rank is maintained in each case (103). It is important to emphasize that cortisol levels can also differ according to the ethological context, and this is related to the fact that aggressive interactions trigger social stress in fish (97). For example, in *O. niloticus* during initial fighting cortisol increases in both opponents (102), but after 5 days of social interactions fish can either show similar levels of stress in homogeneous-sized individuals, or higher levels in dominants than subordinates of heterogeneous-sized group (104).

Taking these species-specific differences and social system characteristics into account, cortisol can be used as a stress indicator in husbandry or experimental conditions. For example, evidence suggests that social isolation during 24 h induces an increase of cortisol levels in *A. nigrofasciata* (63). Environmental enrichment is usually used to improve welfare in housing conditions, creating a more complex and stimulating environment aimed at encouraging the development of natural species-specific behavior (105). In this sense, environment can also modulate isolation-induced stress, since in *O. niloticus* it has been reported that blue light prevents the confinement-induced cortisol response, when compared to green and white light (106). Interestingly, in *O. mossambicus* social isolation elicits a differential increase in cortisol depending on the previous social status of fish, suggesting that it is the perception of social isolation and not the objective isolation itself that triggers the hormonal response to isolation (107). On the other hand, in order to improve space efficiency, fish production usually counts with high stocking densities, which can cause stress in fish. Overcrowding can be identified as a stressor because it can increase aggressive interactions and a concomitant increase in cortisol (91, 97). However, it is important to emphasize that rearing density impacts the HPI axis in a species-specific way according to the social structure of each species. Evidence suggests that while in Atlantic salmon (*Salmo sara*) high-density reared fish show increased expression of stress-related markers and downregulation of innate immune gens, in *O. niloticus* this effect is observed in tilapias held in low densities probably due to increased aggressive interactions (19). Usually body tactile stimulation has a positive effect since it relieves stress and reduces cortisol on highly social animals, including some fish such as the cleaner-client coral-reef fish (108). However, the effect on body tactile stimulation has been tested in *O. niloticus*, reducing aggressive interaction but not plasma cortisol levels (109). This suggests that, even if environmental enrichment with tactile stimulation does not reduce stress in this territorial species, it has a positive effect because it reduces aggressive interactions and it can be used in further studies on fish welfare (109, 110).

Common carp reared in high densities show increased plasma cortisol, glucose and decreased humoral immune response than those in low densities (111). Interestingly, this increased in cortisol can be mitigated by diet enrichment with specific components such as arginine and dietary rosemary leaf powder (111, 112). Another key factor in fish welfare is an appropriate maintenance of water quality, which is essential to reduce stress during transport of ornamental fish. Farmers take different measures to control the stressing effect of transportation (96). Evidence in carps suggest that, even if plasma cortisol and glucose increase after they are distributed in plastic bags for 3 h, high levels of cortisol can be mitigated by diet enrichment with turmeric (113). Moreover, high levels of ammonia in overcrowded stocking are related to food left-overs and unwanted waste and they usually cause an increase in cortisol and glucose, these effects being mitigated by garlic-rich diet (114).

Another strategy to reduce stress responses through diet is by modifying the amino acid composition of the food. L-tryptophan enriched diet has been proven as an effective treatment to reduce stress increase in cortisol in different teleost species, such as *Labeo rohita* (Cypriniformes) (115), *Solea senegalensis* (Pleuronectiformes) (116) and *Totoaba macdonaldi* (Perciformes) (117). In the case of the rainbow trout *Oncorhynchus mykiss*, evidence suggests that L-tryptophan-induced reduction in plasma cortisol levels and suppression of aggressive behavior (118). Interestingly evidence in cichlids suggests not only that L-tryptophan reduces cortisol and aggression in *C. dimerus*, but also that there are no negative impacts on growth parameters (119, 120).

Another important factor affecting stress and fish welfare is light, and evidence in goldfish suggests that the absence of light–dark cycles and feeding cycles increases plasma cortisol levels (121). Regarding environmental enrichment in Cypriniformes, most studies focusing on housing conditions in zebrafish suggest that high cortisol related to stress can be mitigated by environmental enrichment with gravels, artificial plants and objects (122). Environmental enrichment also involves housing tank color. For example, zebrafish which were held in transparent and white tanks increased anxiety-like behaviors compared with zebrafish maintained in black or blue tanks, while blue tanks reduce cortisol levels (123). As already discussed earlier in this section for some cichlid species, isolation in zebrafish evokes anxiety like behaviors and an increase in cortisol levels. Even if anxiety-like behaviors can be mitigated by certain acoustic environmental enrichment, such as exposure to Antonio Vivaldi’s music, isolation-induced cortisol increase persists (124). Considering that plasma or body cortisol measurements represent an invasive technique to fish, a very convenient alternative is to use cortisol dissolved in water as an indicator of fish stress, since there is a significant positive correlation between cortisol released to water and body cortisol (125). Evidence suggests that the pattern of changes in trunk cortisol of stressed zebrafish was also reflected in the water cortisol release rate pattern. Even if the waterborne cortisol release rate can be used as a noninvasive and reliable stress indicator, this methodology needs to be carefully assessed. For example, evidence in zebrafish suggests a higher magnitude of water cortisol release rates than other reported fish species, such as common dentex (*Dentex dentex*), common pandora (*Pagellus erythrinus*), sharpsnout sea bream (*Diplodus puntazzo*), meager (*Argyrosomus regius*), common carp, rainbow trout, common roach (*Rutilus rutilus*), and Atlantic salmon [reviewed in (125)]. Therefore, data on waterborne cortisol needs to be analyzed with caution, since a direct comparison among different studies on different species can be affected by differences in body size, water temperature, metabolic rate, cortisol clearance rate and ventilation rate (125).

Transport and market distribution are also key stressors for ornamental characid species, and efforts are focused on improving the water quality in different ways. In particular, the addition of sodium chloride is one of the most used stress-reducing substances added to transport water for many fish species, including characiforms (126). This is related to the fact electrolytes decrease during the recovery period after hauling, and the addition of salts lower the osmotic gradient between plasma and the environment thus reducing the energy cost of osmoregulation. For example, even if in *Brycon amazonicum* cortisol plasma levels increase immediately after transportation, this is avoided in presence of salt (127). Severe handling events can be also managed by sedating fish with different analgesics, but the appropriate dose is often not well established for different species. Interestingly, essential oils show anesthetic effects for transport (128), while probiotics help to increase fish health during stressful processes such as severe changes in environmental conditions (129). *P. axelrodi* is normally caught in small streams and later transported in plastic boxes before being exported to Europe, Asia, and North America (130). One of the major concerns in this process is mortality during local transportation, which can be attributed to bad management practices and poor water quality leading to disease outbreaks (131). Interestingly, even if cortisol levels increase after transport, this effect is decreased when fish are treated with water-soluble commercial probiotics (132). These results suggest that even if transportation is a major stressor in characiforms commercialization, available tools can help minimize this effect and assure fish welfare.

Similar to evidence discussed in cichlids and Cypriniformes, environmental enrichment and overcrowding also affects characids welfare. Environmental complexity was tested in the checker barb *Oliotius oligolepis*, showing a marked preference for a structured compartment enriched with plants and clay pots (133). Moreover, aggression and foraging activity are used as behavioral welfare indicators to assess the effect of environmental enrichment and group size in *Serrapinnus notomelas*, a Neotropical characin commonly used in the aquarium trade, suggesting that environmental enrichment facilitates foraging activity but not aggression (134). Interestingly, even if many species of Characiformes are most important as ornamental species, to the best of our knowledge there is still no evidence on cortisol as a welfare indicator of stress in captivity conditions.

## 5. Sex steroids and agonistic behavior

The hormonal profile from each individual modulates reproductive and social behavior (e.g., aggression) and, in turn, social interactions with conspecifics also affect the hormonal landscape. In these intertwined and bidirectional effects, steroid levels are usually a result rather than the cause of acquiring a specific social status, since different social contexts modulate hormonal responses (135). In this context, sex steroids are intimately related to aggression and, together with reproductive behavior, they constitute useful information to assess fish welfare.

The social control of reproduction is present in different animal species to a higher or to a lesser extent. Since cichlids are highly social fish, they constitute ideal models to assess how reproduction can be related to different social structures and agonistic interactions (10). This way, dominant and subordinate social status are typically associated to differences in activity throughout the hypothalamic–pituitary-gonadal axis. In *C. dimerus*, territorial dominant males show higher plasma 11-ketotestosterone (11-KT, an important fish androgen) and testosterone (T) levels, but reduced estradiol (E_2_) when compared with non-territorial subordinate males in a social hierarchy (51, 100). In a different ethological context, evidence in dyadic agonistic encounters suggest that winner females show higher E_2_ levels than losers (136). Higher circulating levels of androgens are also associated with reproduction and dominant status in males of *O. niloticus* (137) and *N. pulcher* (48). Similarly, in *A. burtoni*, dominant males present higher plasma T, 11-KT, E_2_ and progesterone (P4) (135). This is paralleled by subordinate males having a low gonadosomatic index, small testes, low fraction of motile sperm, spermatogenesis and sperm density when compared to dominants (138). It is important to emphasize that sex-steroid levels also depend on the ethological context analyzed. As a consequence, when subordinate *A. burtoni* males are given a chance to rise in rank, they rapidly increase plasma levels of T, 11-KT, E_2_ and P_4_ (139). On the other hand, a rapid decrease in plasma androgen levels is observed during social descent from a dominant to subordinate social rank (135). These studies illustrate that social interactions and the establishment of dominance modulate sex-steroid levels.

A nature-based approach of fish welfare implies that when individuals are well, they are able to express their whole behavioral repertoire as if they were in a natural environment. Behavioral integrity can be used as a welfare indicator, since animals should be allowed to perform their natural repertoire of behavior to guarantee their overall welfare (140). To give an example, if reproduction succeeds it means that the artificial environment and housing conditions are adequate for fish welfare (141). As already explained in detail in section 3, cichlid fish have complex reproductive repertoire and, taking this behavioral variability into account, tank maintenance should provide optimal conditions for reproduction to occur, including an adequate sex-ratio, social hierarchy, breeding substrate and environmental enrichment that needs to be settled according to species-specific requirements. For example, while *P. scalare* chooses leaves as a spawning substrate in the natural environment, in captivity fish can lay their eggs in acrylic tubes, suggesting that environmental enrichment can provide an appropriate spawning substrate to facilitate reproduction (142). Water temperature also plays an important role in fish welfare and reproductive patterns. In *O. niloticus* plasma levels of E_2_, cortisol and vitellogenin were significantly reduced under high temperature conditions, suggesting that sex-hormones and steroidogenesis can also be used as welfare indicators to assess water temperature as a stressor (143). As a consequence, reproductive and parental care behavior can also be used as welfare indicators, also taking into account the physiological levels of sex hormones in each species.

Reproduction of ornamental species in captivity can represent an important challenge, and different protocols have been tested according to each species. In the common carp, when spawning in females is induced with Ovaprim or rGnRH, there is an increase in plasma levels of cortisol, T and 20β-dihydroxy-4-pregnen-3-one (DHP), while E_2_ levels are reduced (144). Taking into account that reproductive behavior can be usually induced by administration of sex steroids, further evidence in goldfish shows that administration of 11-KT and T can induce courtship masculinization in females, while treatment with prostaglandin induces feminization of reproductive behavior in males (145). Unfortunately, teleost fish reproduction is drastically affected by endocrine disrupting chemicals present in the aquatic environment (146). Goldfish constitutes a clear example, since certain agents such as Bisphenol affect reproduction at different levels, by inhibiting the expression of genes related to the HPG axis, decreasing 11-KT plasma levels, and disrupting ovarian and testis maturation (147).

Similar to evidence in different cichlids, social behavior in cyprinids is also intimately regulated by sexual steroids. In zebrafish, E_2_ increases shoaling area and social preference, and decreases cortisol levels in male zebrafish (148), while androgens and progestins regulate aggression (149). Taking this into account, social and reproductive behavior can be used as welfare indicators of exposure to different environmental contaminants. There is broad literature revising how reproductive and social behavior are impaired by 17α-ethinylestradiol (EE2), an oral contraceptive that is found in wastewater and effluents (150). Exposure to EE2 has an anxiolytic effect in adult zebrafish (151) and reduces dominant male aggression, impairing dominance and their reproductive success in half of exposed fish (70). Moreover, EE2 reduces females’ court behavior toward males and may have severe implications on the reproductive success of exposed zebrafish populations (152). These studies constitute specific examples on reproductive traits and agonistic behavior as welfare indicators in zebrafish, highlighting how vulnerable teleost fish are to environmental contaminants.

Considering Characiform ornamental species, reproduction in neon tetra *P. innesi* and cardinal tetra *P. axelrodi* is extremely sensitive to environmental conditions and represent interesting challenges for breeding systems. Both species usually prefer soft, slightly acidic water that resembles natural conditions, since water in the streams and rivers of the Amazonian region are poor in nutrients and minerals, but rich in humic acids (153). As a consequence, even if aquarists have been trying to breed them since the mid-20th century, reproduction of these species in captivity is a very difficult task. High market pressure stimulated the development of breeding and rearing methods for tetras (33) but because of its economic relevance there is only limited available data on such information. For example, reproduction in *P. innesi* was successful after a careful protocol with a spawning substrate, increasing temperature up to 25°C, darkness and a specific preparation of acidified water with humic acids (35). In this species, gametes are only produced during few initial spawning events, and even if fish continue to engage in spawning events, resulting offspring is not viable and gametes suffer significant damage after 15–20 days. Also, even if aquarist usually recommend maintaining temperature between 22 and 25°C for this species, evidence on spawning induction suggests that keeping bloodstocks at these temperatures for a prolonged time has a negative effect on spawning and reproduction (154). In *P. axelrodi*, spawning can be induced in laboratory conditions by controlling pH, electric conductivity, temperature at 26°C and the water level with artificial rain simulation (155). Despite various attempts by breeders or researchers to replicate conditions in this experiment, spawning induction was not achieved, probably because in the original protocol experiments were performed with rainwater from the natural habitat of *P. axelrodi* (155). In these species in which reproduction in captivity constitutes such a difficult challenge, the importance of diet on fish maintenance deserves special observation. In *P. axelrodi*, an omnivorous species with a tendency to carnivorous behavior (156), evidence suggests that growth parameters are improved in a diet with 53.5% total protein, and the same effect was reported in *P. innesi* (157). These species represent very extreme examples on the use of reproductive behavior as a welfare indicator of fish husbandry.

## 6. Final remarks

Intensive production of teleost species with ornamental interest faces not only issues related to water quality, diet and sensitivity to environmental conditions, but also high crop densities and stress due to handling and transport, leading to the associated pathologies. Overall, popular practices used in the confinement and farming of these species are affecting reproduction in captivity. This highlights the importance of studying the basic biological requirements and welfare indicators of species with commercial interest, in doing so providing the optimal conditions required by individuals and especially these species whose reproduction is highly sensitive. Considering the above, efforts should focus on studying species-specific social behavior and behavioral indicators such as aggression, courtship and successful reproduction, and also on assessing individual stress and physiological state of the specimens with non-invasive methods, such as cortisol determinations in water. These behavioral and physiological analyses, in turn, can serve as indicators of health, welfare and sexual status of the specimens, helping as a diagnosis to distinguish which points should be further assessed and improved.

## Author contributions

MFS conceived the original design and conception of this manuscript. MFS, LC, and LR wrote the manuscript. All authors contributed to the article and approved the submitted version.

## Funding

This work was supported by the Developing Nations Research Grant from the Animal Behavior Society (ABS), University of Buenos Aires (UBACYT 20020190100010BA), CONICET (PIP 11220200100047CO, PIBAA 28720210100710CO), and the Agencia Nacional de Promocion Científica y Tecnológica (PICT 2021–00043).

## Conflict of interest

The authors declare that the research was conducted in the absence of any commercial or financial relationships that could be construed as a potential conflict of interest.

## Publisher’s note

All claims expressed in this article are solely those of the authors and do not necessarily represent those of their affiliated organizations, or those of the publisher, the editors and the reviewers. Any product that may be evaluated in this article, or claim that may be made by its manufacturer, is not guaranteed or endorsed by the publisher.
